# The Institution-based Network on China–Africa Cooperation for Malaria Elimination: fostering a transcontinental hub for strengthening China–Africa cooperation on malaria control and elimination efforts

**DOI:** 10.1186/s40249-026-01419-8

**Published:** 2026-03-23

**Authors:** Shenning Lu, Wei Ding, Lulu Huang, Longshen Liu, Ning Xiao, Salim Abdulla, Moses Okpeku, Aliou Thiongane, Balla Gibba, Sidzabda Christian Bernard Kompaore, Shan Lv, Shizhu Li, Maru Aregawi Weldedawit, Xiao-Nong Zhou, Duoquan Wang

**Affiliations:** 1 National Institute of Parasitic Diseases, Chinese Center for Disease Control and PreventionChinese Center for Tropical Diseases ResearchNational Key Laboratory of Intelligent Tracking and Forecasting for Infectious Diseases; Key Laboratory of Parasite and Vector Biology, Ministry of HealthWHO Collaborating Centre for Tropical DiseasesNational Center for International Research on Tropical Diseases, Ministry of Science and Technology, Shanghai, 200025 China; 2 Ifakara Health Institute, Dar es Salaam, Tanzania; 3 School of Agriculture and Sciences, University of KwaZulu-Natal, Durban, South Africa; 4 Programme National de Lutte Contre le Paludisme, Ministère de la Santé et de l’Action Sociale, Fann Résidence, Dakar, Sénégal; 5 National Malaria Control Program, Ministry of Health, Banjul, The Gambia; 6 Secrétaire Permanent Pour L’élimination du Paludisme, Ministry of Health, Ouagadougou, Burkina Faso; 7 School of Global Health, Chinese Center for Tropical Diseases Research, Shanghai Jiao Tong University School of Medicine, Shanghai, 200025 China; 8 World Health Organization, Geneva, Switzerland

**Keywords:** Malaria, Network, China-Africa collaborative initiatives, Institution-based Network on China–Africa cooperation for malaria elimination

## Abstract

Malaria remains a significant global health challenge, particularly in Africa, which continues to bear the greatest burden. The transboundary nature of malaria necessitates regional collaboration, yet transcontinental knowledge-sharing mechanisms remain limited. The World Health Organization’s (WHO) certification of China as malaria-free in 2021 presents an opportunity to leverage its expertise, cost-effective products, and elimination strategies to support Africa’s malaria control efforts. The Institution-based Network on China-Africa Cooperation for Malaria Elimination (INCAM) was established in 2018 to bridge this gap, fostering cross-continental collaboration through knowledge exchange, product innovation, and policy advocacy. Between 2019 and 2025, INCAM has convened six fora, facilitated to promote the adaptation of China’s anti-malaria practices to African contexts. These fora promoted the exploration of demand-driven research, the development of innovative anti-malarial products, and the advancement of evidence-based policies. INCAM exemplifies the potential of China-Africa collaboration to accelerate malaria control and elimination in Africa through solutions tailored to local contexts. To sustain this progress, there is an urgent need for stronger Public–Private-Academic Partnerships, and enhanced capacity-building programs. By addressing these gaps, INCAM can maximize its role as a leading transcontinental hub for malaria control and elimination for China and Africa.

**Graphical abstract:**

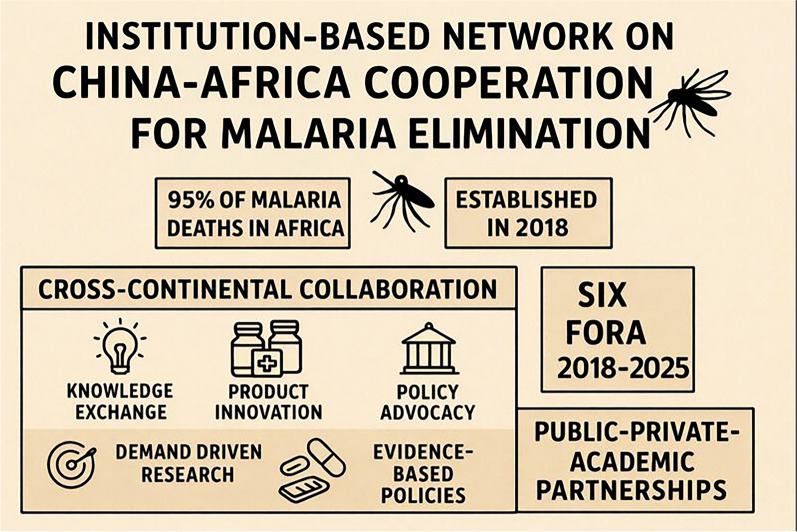

## Background

Malaria’s transboundary nature, driven by human migration, shared ecological zones, and cross-border trade, demands coordinated regional action. These dynamics facilitate parasite spread across borders, particularly in regions where health systems, policy frameworks, and political priorities diverge. Such fragmentation impedes the alignment of surveillance and response efforts, as well as the efficient mobilization and allocation of resources. Recognizing these challenges, the World Health Organization (WHO) Global Technical Strategy for Malaria 2016–2030 explicitly calls to “deepen regional collaboration” to achieve malaria elimination [1]. Successful examples that embody this approach include the Asia–Pacific Malaria Elimination Network (APMEN) which has expanded from 10 to 16 malaria-endemic countries in Greater Mekong Subregion since 2009 [2], the Asia–Pacific Leaders Malaria Alliance(APLMA), and the Greater Mekong Subregion Malaria Elimination (MME) programme. In Africa, the Malaria Elimination 8 (E8) initiative unites eight southern African countries to coordinate cross-border surveillance and resource pooling [3], while the Africa Leaders Malaria Alliance(ALMA) galvanizes political commitment at the head-of-state level to harmonize elimination programs [4]. While these validate regional coordination as essential, a critical gap remains in the structured mechanisms for sharing best practices and lessons learned across continents, particularly between elimination-certified and high-burden regions.

Global progress against malaria remains fragile, particularly in the WHO African Region. According to WHO’s World Malaria Report 2025, there were 282 million cases and 610,000 deaths in 2024, with the WHO African Region accounted for 94% of global malaria cases and 95% of malaria deaths in 2024 [5]. Key persisting gaps in malaria control in Africa include weak surveillance systems, limited access to novel tools, and insufficient sustainable financing and digital technologies for data-driven management [6]. China’s malaria elimination certification by WHO in 2021 is seen as a strategic advantage to leverage its antimalarial products, experience, and expertise in Africa [7]. China’s experience includes the “1–3-7” surveillance and response strategy, tailored interventions based on local contexts, and a whole-of-government approach involving multi-sector coordination and community engagement [7]. However, due to significant differences in epidemiological profiles, health system capacities, and ecological contexts, China’s strategies are not directly applicable to high-transmission settings in sub-Saharan Africa. Instead, they provide valuable principles—such as rigorous surveillance, phased-specific strategy, stratification-based guidance and community mobilization—that can inspire and inform context-appropriate adaptations. Effective malaria control in these regions requires a comprehensive approach, as conceptualized under Integrated Vector and Parasite Management, which underscores the need for multi-faceted, locally tailored interventions [8]. Furthermore, the availability of cost-effective anti-malaria products, mainly the medicines, diagnostics, and vector control tools, meeting the WHO pre-qualification standards can facilitate market expansion into Africa or support the localized manufacturing. China consistently supports Africa in malaria control and elimination through bilateral and multilateral cooperation. The lynchpin of China-Africa malaria cooperation is the Forum on China-Africa Cooperation (FOCAC) which has spawn a series of flagship projects (Table 1).

**Table 1:** An overview of the China-Africa malaria cooperation projects

Project	Duration	Partners
Fast Elimination of Malaria through Source Eradication project in Comoros and São Tomé and Príncipe	now 2017-now	Guangzhou University of Chinese medicine, Comoros Ministry of Health, São Tomé and Príncipe Ministry of Health
China–UK–Tanzania Pilot Project on Malaria Control	2015–2018	UK Department for International Development, Ifakara Health Institute(IHI), National Institute of Parasitic Diseases at Chinese Center for Disease Control and Prevention (NIPD at China CDC)
China–Tanzania Demonstration Project on Malaria Control	2018–2023	Gates Foundation, Tanzania National Malaria Control Programme (NMCP), IHI, NIPD, World Health Organization (WHO) Tanzania Office, Harvard University, Tsinghua University
China-Africa Cooperation Project on Malaria Control	2020–2024	China National Health Commision, China CDC, NIPD at China CDC, Henan CDC, Shandong Institute of Parasitic Disease, Tanzania NMCP, Zambia National Malaria Elimination Center
Strengthening the capacity of surveillance and response in selected African countries to control Malaria in different settings by China-Africa networking control Malaria in different settings China-Africa networking	2022–2024	WHO Global Malaria Programme (GMP), WHO TDR (the Special Programme for Research and Training in Tropical Diseases), WHO Country Offices in Senegal, Zambia and Burkina Faso, National Malaria Programmes in Senegal, Zambia and Burkina Faso
Gambia Accelerating Malaria Elimination	2024–2026	China International Development Cooperation Agency, International Federation of Red Cross and Red Crescent Societies, Gambia Red Cross Society, Gambia NMCP, NIPD

Previous studies have synthesized global and regional malaria initiatives, as well as China-Africa health cooperation on neglected diseases, like schistosomiasis [9]. However, there has been limited exploration of dedicated, institution-based mechanisms for sustained China-Africa malaria cooperation. This study addresses this gap by examining the Institution-based Network on China-Africa Cooperation for Malaria Elimination (INCAM), launched at the 2018 China-Africa Health Cooperation High-Level Meeting. We provide a comprehensive overview of the INCAM network by exploring its founding context, progress, challenges, and recommendations, positioning it as a complementary mechanism that supports both intra-African regional collaboration and WHO’s global leadership.

## Main text

### Establishment of INCAM

During the 2018 High-Level Meeting on China-Africa Health Cooperation, the INCAM was officially proposed through a collaborative effort among multiple leading institutions. This initiative built upon existing foundations, including prior Memoranda of Understanding (MoUs) signed between China's National Institute of Parasitic Diseases (NIPD) and key African research entities such as Sudan's Blue Nile National Institute for Communicable Diseases and South Africa's Center for Sustainable Malaria Control, University of Pretoria. The founding consortium comprised the National Institute of Parasitic Diseases, Chinese Center for Disease Control and Prevention (China CDC); Université des Montagnes (Cameroon); Ethiopian Public Health Institute (Ethiopia); Directorate of Disease Prevention and Control, Ministry of Health and Sanitation (Sierra Leone); Ifakara Health Institute (Tanzania); University of Namibia (Namibia); and National Malaria Elimination Centre (Zambia).

The network expanded significantly in 2019 when two additional institutions—the Institute for Research in Health Sciences/Crun (Burkina Faso) and the Malaria Research and Control Center (Côte d'Ivoire)—joined via MoUs, bringing membership to nine countries. In subsequent years, INCAM has continued to grow through its annual conferences, with new national representative institutions joining and signing cooperation agreements. Notable additions include Senegal's Programme National de Lutte contre le Paludisme and Gambia’s National Malaria Control Programme. Through these expanding partnerships, INCAM’s institutional collaboration network now comprehensively covers 13 countries across China and Africa, creating a robust platform for China-Africa malaria control cooperation that extends beyond the original founding members to include both historically engaged and newly participating nations.

**Fig. 1:**
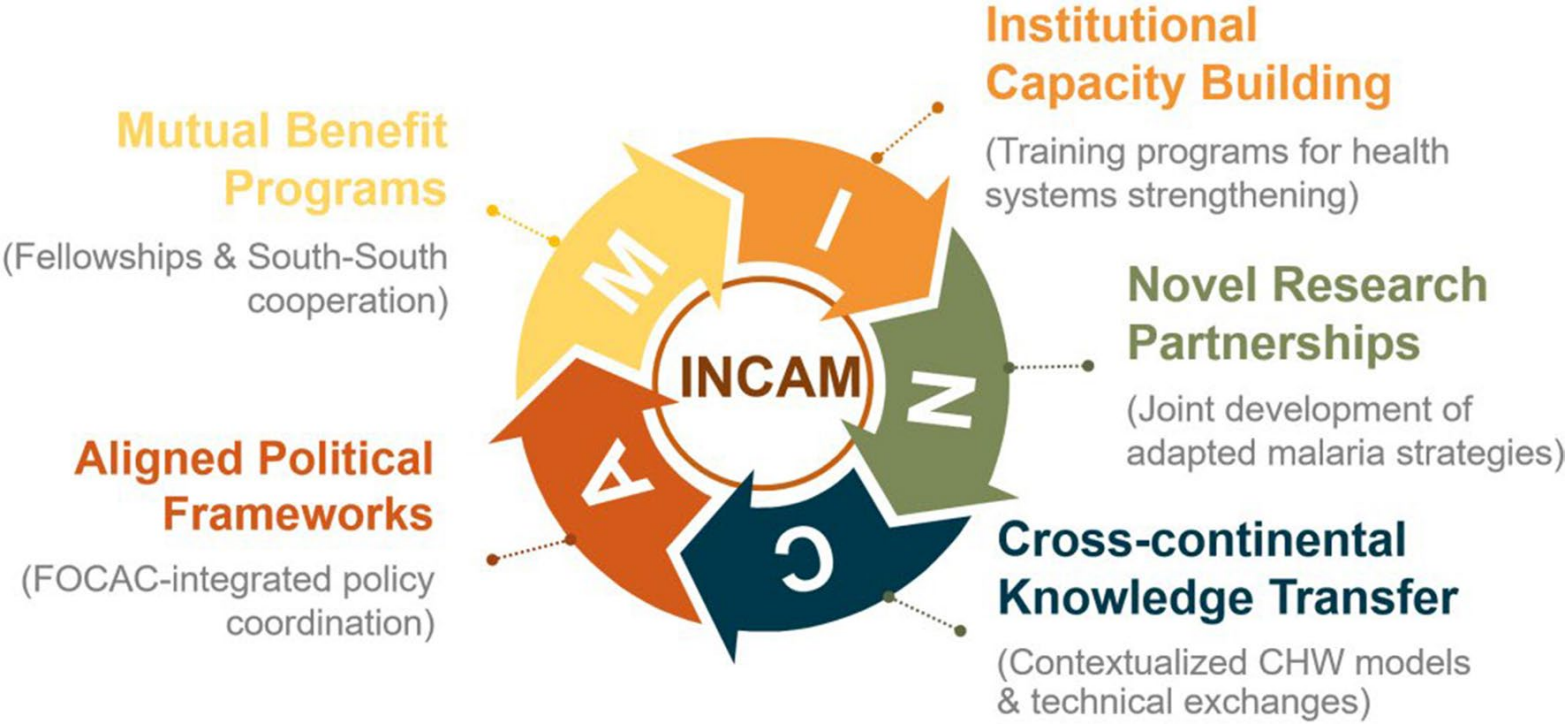
Institution-based Network on China-Africa Cooperation for Malaria Elimination five core pillars. INCAM: Institution-based Network on China-Africa Cooperation for Malaria Elimination; CHW: community health worker; FOCAC: Forum on China-Africa Cooperation

INCAM aims to promote transcontinental synergy through five core objectives (Fig. 1). First, it focuses on institutional capacity building by strengthening health systems through targeted training programs designed to enhance operational effectiveness. Second, it fosters novel research partnerships by promoting collaborative initiatives that adapt China’s evidence-based malaria strategies to the African context. The network also emphasizes cross-continental knowledge transfer, facilitating the exchange of best practices and expertise, particularly through the adaptation of community health worker models for malaria control. Additionally, INCAM seeks to ensure aligned political frameworks by driving policy integration through FOCAC-driven dialogues, aligning malaria control efforts with broader political and international cooperation initiatives. Finally, the network aims to implement mutual benefit programs that support South-South cooperation, including fellowship opportunities to promote cross-cultural learning and professional development. To realize this mandate, INCAM converges efforts across four cooperation domains: technical integration of China’s cost-adaptive diagnostics/vector control products; unified surveillance tools harmonizing Pf/Pv resistance monitoring across borders; knowledge commons co-building open-access outbreak alert platforms bridging Chinese AI prediction models with African field networks; and human capital synergy. Critically, INCAM catalyzes cross-domain integration—where surveillance data fuels research priorities, capacity-building enables technology adoption, and policy dialogues anchor equitable resource allocation.

### Main achievement

Based on the five core objectives established at its inception and the corresponding progress made, we can observe that INCAM has achieved notable advancements in several key areas (Table 2). Specifically:

**Table 2:** Institution-based Network on China-Africa Cooperation for Malaria Elimination fora schedule and basic information

Fora	Year/location	Format	Participants	Key agenda items	Major outputs
1st	2019/shanghai	Offline	55 + delegates (7 countries)	China’s 60-year integrated malaria-schistosomiasis control trajectory and the “1–3-7” strategy; malaria situation and bottlenecks from Ghana, Kenya, Malawi, Nigeria, Tanzania and Côte d’Ivoire; field progress of the China-Tanzania demonstration project	Signing of INCAM MOU China-Africa Malaria Cooperation Framework
2nd	2020/Shanghai	Hybrid	20 + delegates; 1000 + virtual attendees (10 countries)	COVID-19 impact modelling on malaria transmission in Africa; Malaria elimination in China; country updates from South Africa, Zambia and Cameroon	establishment of INCAM rotating-chair mechanism academic-committee blueprint
3rd	2021/Haikou	Hybrid	35 + delegates; 250 + virtual attendees (10 countries)	Multisectoral Approaches against Vector-Borne Diseases in Africa; country updates from Burkina Faso, Sierra Leone and Namibia; Gates Foundation's partnership model with Chinese companies	Action plan for INCAM working mechanism
4th	2022/Haikou	Hybrid	30 + experts (9 African countries)	Multisectoral Approaches against Vector-Borne Diseases in Africa; country updates from Ethiopia, Sierra Leone and South Africa; Framework indicator for distilling China anti-malaria experiences	Roadmap to scale up “1,7-mRCTR” approach in African settings
5th	2024/Shanghai	Hybrid	30 + delegates (WHO GMP, African NMCPs, Gates Foundation,PATH, CHAI, Universities, China CDC, Provincial CDCs, and Chinese enterprises)	country updates from Ethiopia, Senegal and South Africa; implication from 1, 7-mRCTR approach successful in the Southern Tanzania	Sustaining Strategies for China-Africa Malaria Cooperation
6th	2025/Changsha	Hybrid	50 + delegates (WHO GMP, African NMCPs, Gates Foundation,PATH, CHAI, Universities, China CDC, Provincial CDCs, and Chinese enterprises)	country updates from Gambia and Zambia; Chinese Innovative Malaria RDTs	China’s potential contributions in products research and development to malaria control in Africa

WHO GMP: World Health Organization Global Malaria Programme; NMCP: National Malaria Control Programme; PATH: Program for Appropriate Technology in Health; CHAI: Clinton Health Access Initiative; CDC: Center for Disease Control and Prevention; 1, 7-mRCTR: 1,7-malaria Reactive Community-based Testing and Response; RDT: Rapid Dignostic Test; INCAM: Institution-based Network on China-Africa Cooperation for Malaria Elimination

1. Promoting equitable health cooperation: INCAM has successfully fostered health cooperation between China and Africa through six fora (2019–2025). Since its inception, INCAM has provided platform for partners to sign and renew a series of cooperation agreements, among 13 countries, laying the foundation for subsequent cooperation. Additionally, INCAM has promoted approximately 10 bilateral and multilateral cooperation projects to be proposed or implemented. However, despite these achievements, the lack of consistent, long-term funding remains a primary constraint, affecting the continuous implementation and expansion of projects.
2. Developing joint research: INCAM has made significant progress in joint research, particularly in sharing China's “1–3-7” surveillance and response approach (reporting within 1 day, case investigation within 3 days, and foci response within 7 days) and developing the 1,7-malaria Reactive Community-based Testing and Response (“1,7-mRCTR”) approach which is more suitable for African context during malaria control. The malaria prevalence declined by 81% in pilot areas and by an average of 17% across three moderate- to high-transmission districts, illustrating its potential for scalable adoption in similar African settings [10]. Through multiple fora and workshops, INCAM has facilitated cooperation between Chinese and African research institutions, advancing evidence-based research on malaria control and elimination. For example, the fourth forum held in Haikou, China in 2022 proposed a framework for applying the “1,7-mRCTR” approach in Africa. However, the sustainability and depth of these research activities are limited due to the unstable funding and limited resources, affecting further translation and application of research results.
3. Strengthening institutional capacity: INCAM has significantly enhanced the institutional capacity of member countries through training and capacity-building activities. For example, the third forum held in Haikou, China in 2021 developed an action plan for the INCAM working mechanism, promoting technical exchanges and capacity-building among member countries. Additionally, INCAM has shared China's experience to member countries in Africa through the community health worker (CHW) model, enhancing local capacity to combat malaria. The sustainability of these capacity-building activities is challenged due to the lack of long-term funding and professional personnel.
4. Transferring contextualized lessons: INCAM has transferred China's CHW experience to Africa through multiple fora and field projects. For example, the fifth forum held in Shanghai, China in 2024 discussed how to promote the “1,7-mRCTR” approach in African countries and developed corresponding sustainable strategies. The absence of formal government commitment and support for Public–Private-Academic Partnerships limits the potential for scaling up these transfer initiatives.
5. Enforcing mutual benefit via cross-cultural fellowships: INCAM has promoted personnel exchanges and capacity-building between China and Africa through cross-cultural fellowship programs. Through INCAM, researchers in the member countries had opportunities to study and train in China, while also offering Chinese experts the experience of working in Africa. However, the previous exchange and training programmes rely on the funding sources from NIPD, so the scale and impact of these training programs are limited.

### Key outcome

Collectively, the INCAM fora have yielded three significant outcomes:

#### Knowledge/expertise sharing

INCAM fora established a transcontinental knowledge hub that redefined traditional technical assistance paradigms. By sharing WHO's global malaria updates with African members contributing context-specific epidemiological progress and challenges, the network enabled critical information exchange and dynamic adaptation of China's elimination models. Crucially, Chinese malaria intervention model like the “1-3-7” surveillance and response were systematically recalibrated for African realities, exemplified by Tanzania’s community-led 1,7-mRCTR approach [11]. Concurrently, China-Africa malaria initiatives from the government level as well as global stakeholders such as IFRC and Gates Foundation are shared, helping targeted African countries and technical institutions to better identify the way-forward in addressing gaps in malaria control and elimination efforts [12].

#### Product promotion: catalyzing demand-driven innovation

INCAM fundamentally reengineered product development into a demand-driven co-creation cycle initiated by African national malaria programmes and implementation institutions articulating critical needs which directly informed Chinese anti-malaria manufacturers’ R&D priorities [13]. Companies subsequently redesigned products to meet these specifications, moving beyond generic exports toward context-responsive innovation. This market alignment was technically anchored by China’s research institutions, which provided vital scientific scaffolding: validating prototypes against locally prevalent *P. falciparum* strains, optimizing supply chains tailored to Africa’s infrastructural needs, and exploring co-developing testing protocols with African regulators to ensure compliance. A key objective of this co-development model is to design products that are aligned with WHO prequalification requirements or robust national regulatory standards from the outset. The pathway then deliberately transitioned toward localized ownership through strategic joint ventures that established in-region manufacturing capabilities while transferring technical expertise. This shift is not merely about technology transfer, but embodies a longer-term vision to catalyze African-led production ecosystems, informed by China’s experience in achieving commodity security through graduated industrial capacity building and strategic policy support. Crucially, INCAM’s Public–Private-Academic Partnership (PPAP) framework hopes to mobilize African investment and policy incentives to sustain these ventures, completing the transformation from externally dependent procurement to African-governed production ecosystems. This end-to-end integration—where African demand sets the agenda, Chinese industry adapts solutions, academic bridges validate feasibility, localization leverages regional assets, and institutional mechanisms secure sovereign ownership—transcends transactional commerce to establish a self-reinforcing model for equitable health technology justice.

#### Policy advocacy: institutionalizing evidence-to-policy pathways

INCAM’s advocacy efficacy stemmed from its embedded policy translation architecture, where structured forum dialogues converted field innovations into scalable intervention model. A paradigmatic case is the China-Tanzania 1,7-mRCTR approach: first piloted in Rufiji District in Southern Tanzania, then demonstrated in two more districts, and later scaled up in Zambia, Burkina Faso and Senegal [14]. Crucially, INCAM engineered a replicable pipeline for such innovations: 1) Proof-of-concept workshops where African projects collaborators stress-tested strategies; 2) Technical deep dives co-led by Chinese implementers and African epidemiologists; 3) African National Malaria Programmes and international experts such as from WHO GMP recognized the promising results and committed to further validate, so as to systematically elevated into national strategic plans.

### Challenges and pathways

**Fig. 2:**
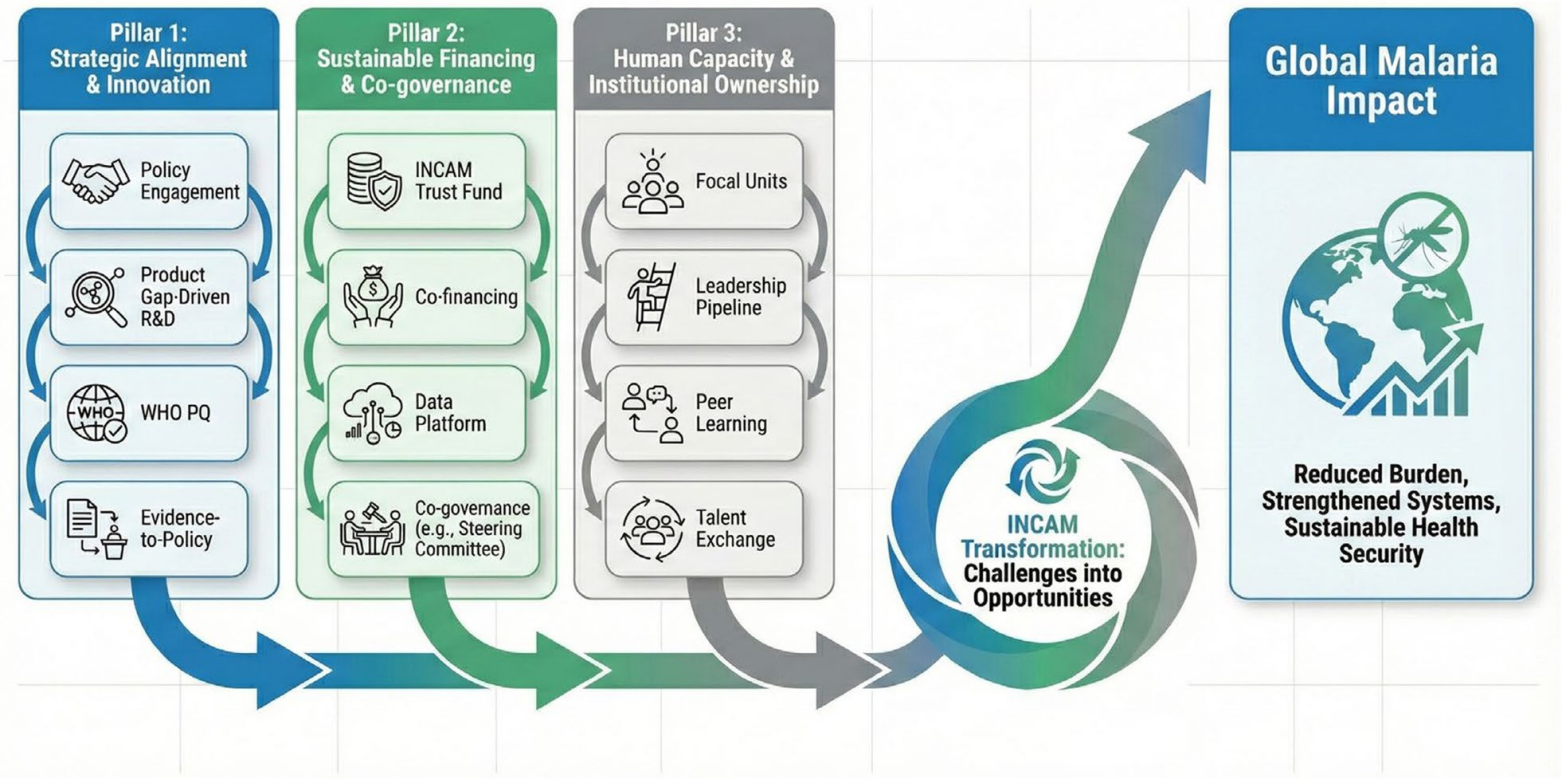
Institution-based Network on China-Africa Cooperation for Malaria Elimination strategic advancement framework

Despite progress, INCAM faces critical hurdles including unable to secure consistent, long-term funding remains a primary constraint, hindering project implementation, research activities, and network sustainability. Limited dedicated personnel within member institutions for INCAM coordination, project management, and research collaboration impacts the network's operational efficiency and depth of engagement. Enhanced commitment and practical support from governments, particularly regarding the PPAP, are essential for scaling interventions and ensuring policy alignment. To transform these challenges into opportunities and unlock INCAM's full potential, we propose (Fig. 2):

1. Enhance PPAP support: INCAM should strengthen its PPAP initiatives by actively engaging policymakers from China and Africa to secure high-level political commitment. This includes securing policy advocacy and guarantees from national and provincial CDCs for technical assistance for on-ground malaria interventions in Africa. INCAM also needs to encourage malaria-product enterprises to understand the latest demands of African malaria-control programs, especially product gaps—such as long-lasting insecticide-treated nets, drone-based larviciding, Point-of-Care Testing (POCT) diagnostics. By integrating their strengths into research and development(R&D) efforts, these enterprises can contribute to Africa's malaria-control endeavors while facilitating the globalization of Chinese products, such as achieving WHO Prequalification and local manufacturing. On the academic front, INCAM should enhance the theoretical refinement of malaria-control research and the translation of evidence-to-policy processes.
2. Establish sustainable funding and operation mechanisms: To ensure INCAM’s long-term functionality and integration into the global malaria financing ecosystem, a dedicated financing and institutional infrastructure is essential. An INCAM Trust Fund should be established, capitalized by anchor grants from Chinese and African governments and possible allocations from the intentional donors. It should be designed to complement with major international financiers like the Global Fund and the World Bank, by providing co-financing for joint China-Africa proposals to larger pools, funding preparatory work to enhance members’ competitiveness for major grants, and supporting downstream activities (e.g., localization, operational research) that fall outside the scope of traditional grants. A lean secretariat will host a bilingual/multilingual data-sharing platform that tracks every China-Africa malaria project, maps unmet product and capacity needs, and guides members step-by-step through grant calls and application procedures. Governance will be reinforced through a Steering Committee comprising high-level representatives from China, Africa, and donors, and a Technical Advisory Group to adjust strategies, which should guarantee at least 50% membership and voting rights for African partners. This governance model is fundamental to transforming INCAM from a network facilitated by China into a truly co-owned platform where African priorities directly shape the agenda and resource allocation, thereby dismantling the traditional donor-recipient paradigm.
3. Strengthen human capacity: INCAM should commit dedicated teams across member institutions to enhance the capabilities of personnel involved in China-Africa malaria control cooperation, by promoting a systemic empowerment model. This involves establishing dedicated focal units within African institutions to ensure continuity and local ownership of the cooperation agenda. The China-Africa Malaria Fellowship should evolve into a leadership pipeline, combining advanced training in China with applied, mentor-supported projects at home to cultivate a sustainable cohort of local experts. Furthermore, INCAM can facilitate peer-to-peer learning networks (via digital hubs and workshops) where African and Chinese practitioners jointly solve contextual challenges, reducing recurring needs for external technical assistance. Additionally, INCAM could establish a talent exchange program, where key INCAM members are temporarily assigned to donor institutions or specialized malaria control agencies in China and Africa. This would allow them to gain hands-on experience, understand real—time needs and operational mechanisms, and enhance their professional and communication skills. By building a well—trained and well—connected workforce, INCAM can ensure sustained and effective collaboration in malaria control efforts.

## Conclusions

INCAM has evolved into a functional platform for cross-continental collaboration on malaria elimination since its inception, facilitating knowledge transfer, product development, and policy advocacy. Through six consecutive fora (2019–2025), the network has advanced its core mission by establishing knowledge-exchange ecosystems that adapt China malaria elimination methodologies (e.g., 1,7-mRCTR approach) to African contexts, initiating demand-driven innovation pathways where African priorities directly shape Chinese product R&D and localization initiatives and helping to build evidence-to-policy pipelines. Given the frequent co-endemicity of malaria and schistosomiasis in many areas, there is further potential for operational synergy between INCAM and the parallel Institution-based Network on China-Africa Cooperation for Schistosomiasis Elimination (INCAS). Despite these achievements, INCAM's long-term sustainability faces critical constraints: funding instability jeopardizing network continuity, human resource gaps limiting operational capacity and insufficient government anchoring of Public–Private-Academic Partnerships (PPAP). To realize its full potential, INCAM requires a stronger PPAP model, institutionalized financing mechanisms, and enhanced cross-border human capacity building programs. Addressing these imperatives will be crucial to the network's capacity to leverage China’s malaria control and elimination experience and products to accelerate malaria control in Africa.

## Data Availability

All data generated or analysed during this study are included in this published article and its supplementary information files.

## List of abbreviations

INCAM: Institution-based Network on China-Africa Cooperation for Malaria Elimination
WHO: World Health Organization
APMEN: Asia–Pacific Malaria Elimination Network
APLMA: Asia–Pacific Leaders Malaria Alliance
MME: Greater Mekong Subregion Malaria Elimination
E8: Malaria Elimination 8
ALMA: Africa Leaders Malaria Alliance
MoUs: Memoranda of Understanding
NMCP: National Malaria Control Programme
FOCAC: China–Africa malaria cooperation is the Forum on China–Africa Cooperation
IHI: Ifakara Health Institute
NIPD: National Institute of Parasitic Diseases
China CDC: Chinese Center for Disease Control and Prevention
GMP: Global Malaria Programme
IFRC: International Federation of Red Cross and Red Crescent Societies
PPAP: Public–Private-Academic Partnership
